# Creating and sensing asymmetric lipid distributions throughout the cell

**DOI:** 10.1042/ETLS20220028

**Published:** 2022-11-14

**Authors:** Guillaume Drin

**Affiliations:** Université Côte d'Azur, CNRS, Institut de Pharmacologie Moléculaire et Cellulaire (IPMC), Valbonne, France

**Keywords:** asymmetry, cellular membrane, lipid synthesis, lipid transfer, membrane contact sites

## Abstract

A key feature of eukaryotic cells is the asymmetric distribution of lipids along their secretory pathway. Because of the biological significance of these asymmetries, it is crucial to define the mechanisms which create them. Extensive studies have led to the identification of lipid transfer proteins (LTPs) that work with lipid-synthesizing enzymes to carry lipids between two distinct membranes in a directional manner, and are thus able to create asymmetries in lipid distribution throughout the cell. These networks are often in contact sites where two organelle membranes are in close proximity for reasons we have only recently started to understand. A question is whether these networks transfer lipids *en masse* within the cells or adjust the lipid composition of organelle membranes. Finally, recent data have confirmed that some networks organized around LTPs do not generate lipid asymmetries between membranes but sense them and rectify the lipid content of the cell.

## Key lipids are asymmetrically distributed in the cell

**Figure 1.**
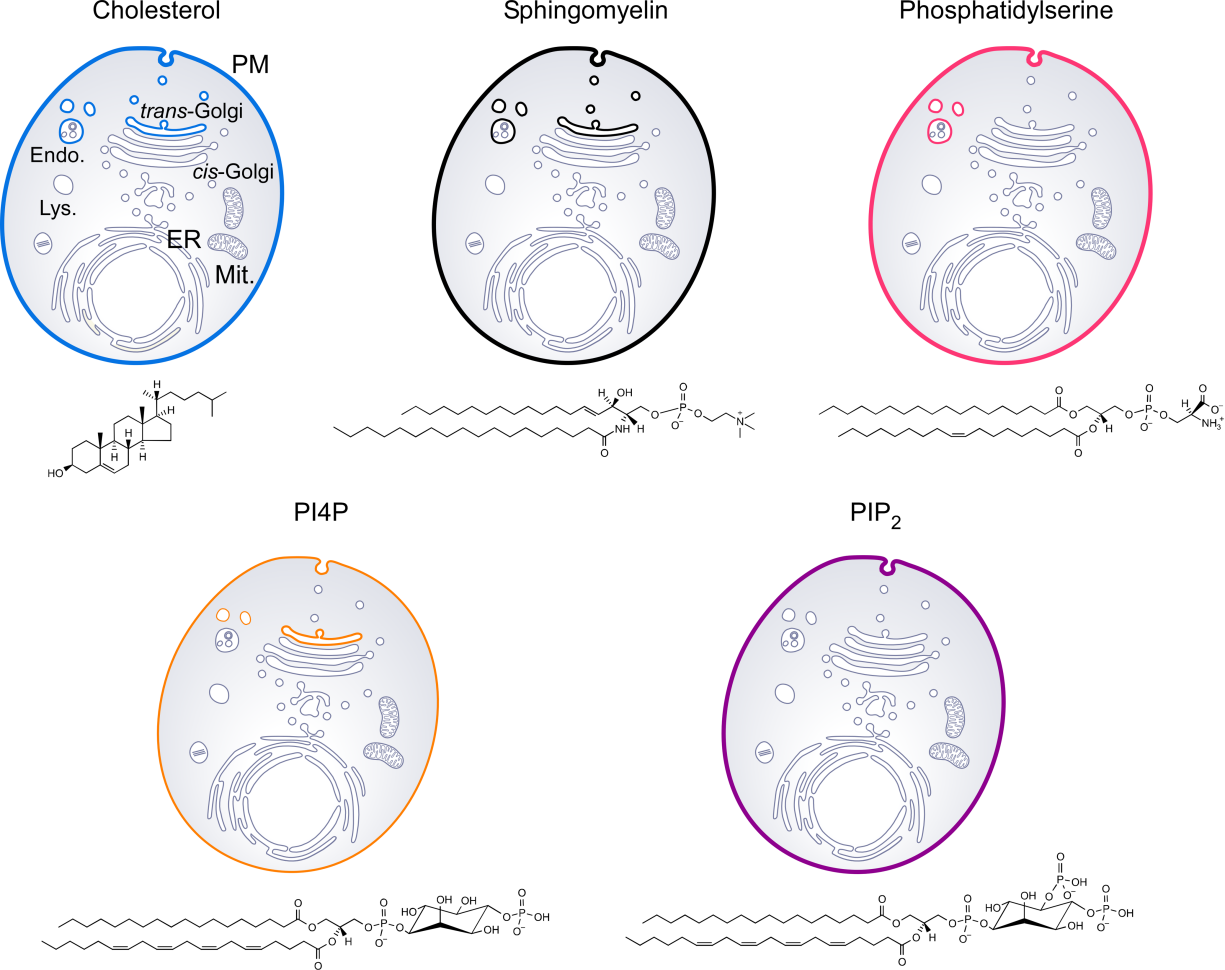
Asymmetric distribution of major lipids in the cell. Sterols like cholesterol are rigid and highly hydrophobic molecules made of four fused rings that possess an alcohol function and a short carbon chain. Sphingolipids, like sphingomyelin, and phospholipids (PS, PI4P and PIP_2_) are composed of a polar head and two carbon chains, which are hydrophobic and flexible. In this representation of a human cell, membranes that contain most of each type of lipid are coloured (blue: cholesterol; black: sphingomyelin; pink-red: phosphatidylserine; orange: PI4P; purple: PIP_2_). The asymmetries in sterol, sphingolipid, PS and phosphoinositide distributions overlap, which allows the ER/cis-Golgi and trans-Golgi/PM regions to constitute two membrane territories in the cell with contrasting features. Endo: endosome; Lys: lysosome ; Mit: mitochondrion; PM: plasma membrane.

Eukaryotic cells have a compartmentalized architecture based on membranes composed of lipids and proteins. These lipids are very diverse but mostly belong to three classes: phospholipids, sphingolipids and sterols. In addition to serving as building-blocks — they organize into bilayers to constitute the cellular membranes — lipids play functional roles through interactions with specialized proteins. All are precisely distributed between the plasma membrane (PM), which is the limiting membrane of the cell, and the membranes that delimit its internal compartments i.e. organelles like the endoplasmic reticulum (ER) or Golgi apparatus [1,2]. A general hallmark of eukaryotic cells is the asymmetric distribution of different lipids along the secretory pathway, i.e. between a region corresponding to the ER and cis-Golgi and a region comprising the trans-Golgi, endosomes and the PM (Figure 1). This defines two membrane territories with different lipid composition and contrasting features, in terms of both fluidity, thickness and surface charge [1,2]. As a consequence, these territories can harbour specific molecular mechanisms that ensure local signalling pathways, protein exchange between organelles via particular vesicular trafficking routes, and the organization of the cortical cytoskeleton. What are the nature and specific roles of the lipids that are asymmetrically distributed?

Known examples are sterols and sphingolipids, which represent, respectively, ∼12–20% and 7.5–10% of membrane lipids in eukaryotic cells [3,4]. Sterol accounts for less than 5% of lipids in the ER but 10% in the trans-Golgi membrane and up to 40% in the PM [5–8]. Similar concentration gradients of sphingolipids have been observed [5,8]. This co-distribution of sterol and sphingolipids is critical for the cells, notably because, as they associate preferentially, these lipids increase the rigidity and thickness of membranes in which they are abundant [9]. This allows the PM to form an impermeable barrier between the cell interior and the external milieu but also to have a precise inner organization [10].

Another lipid is phosphatidylserine (PS), a negatively charged phospholipid species that accounts for 2–10% of cellular lipids [3,4,11]. In the ER membrane, its proportion among lipids is 3–7%, and is multiplied by 4–5 in the PM [6,12,13]. PS mostly concentrates in the inner layer of this membrane where it contributes negative charges and recruits cytosolic proteins with signalling functions via electrostatic interactions [14,15]. The last examples are phosphoinositides, a group of seven phospholipid species that represent ∼1–2% of lipids and derive from phosphatidylinositol (PI). They are absent from the ER and are mostly found in the trans-Golgi and endosomal membrane, and the PM, where they play pivotal roles in vesicular trafficking, cytoskeleton organization or signalling pathways [16,17].

Considerable efforts have been made to explain the origins of these asymmetries in lipid distribution. One goal is to understand how lipid species are enriched in the trans-Golgi or PM while being partially or entirely created in the ER. Another aim is to define how these asymmetries are maintained despite the bulk exchange of lipids between organelles by vesicular trafficking, but also fast lipid enzymatic conversion and uptake.

## How are intracellular lipid asymmetries created?

Current data suggest that lipid asymmetry within cells is at least partially created by small networks composed of lipid-synthesizing enzymes, located in distinct cellular membranes, and lipid transfer proteins (LTPs) that flow lipids between these membranes. LTPs belong to diverse families, but all have a domain with a cavity to encapsulate one or several lipids [18,19]. This allows these proteins to extract lipids from an organelle membrane and shield these hydrophobic molecules from the aqueous medium (i.e. cytosol) for delivery to a second membrane. Remarkably, these networks are often organized around contact sites where the membranes of two distinct organelles are less than 30 nm apart [20], which is an extremely short distance at the cellular scale.

**Figure 2.**
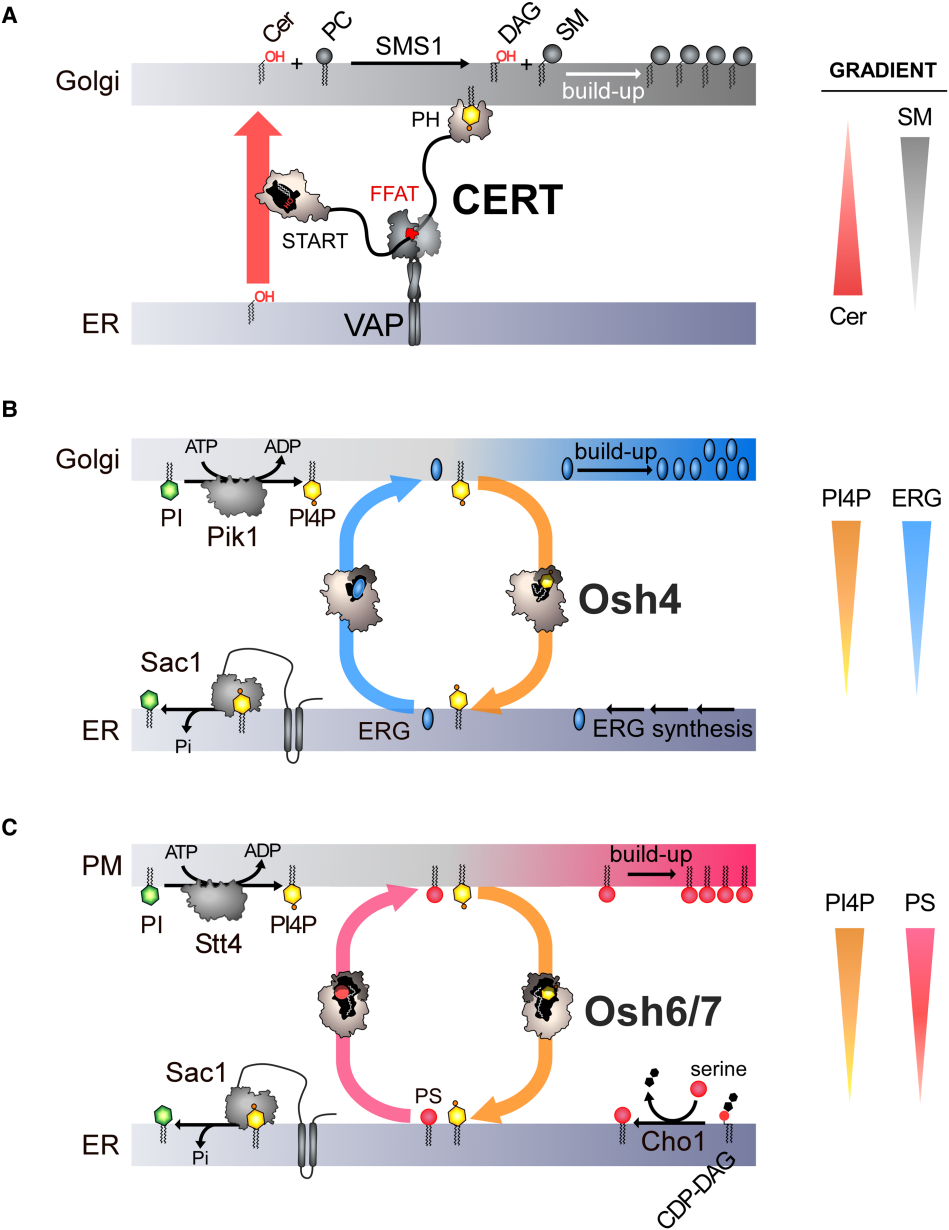
Protein networks that create an asymmetric distribution of lipids between membranes. (**a**) CERT engages ER–Golgi contacts by associating with PI4P present in the trans-Golgi membrane via its PH domain and by binding to the ER-resident VAP receptor via an FFAT motif. Then, CERT carries ceramide (Cer) by means of its START domain between the two organelle membranes. Ceramide is converted into sphingomyelin (SM) by the Golgi SMS1 enzyme. Phosphatidylcholine (PC) is used as the donor of the phosphocholine head group in this catalyzed reaction in which diacylglycerol (DAG) is also produced. This metabolic trapping event enables an ER-to-Golgi transfer of ceramide and the creation of an asymmetry in sphingolipid distribution. (**b**) In yeast, ergosterol (ERG) is produced in the ER. In this model, Osh4 conveys ergosterol from the ER to the trans-Golgi, and PI4P along the opposite direction. A PI 4-kinase called Pik1 transforms PI into PI4P in an ATP-dependent manner in the Golgi membrane, while Sac1 hydrolyzes PI4P at the ER thereby maintaining a PI4P gradient at the ER/Golgi interface. This allows for multiple cycles of exchange and thus the build-up of ergosterol in the Golgi membrane at the expense of the ER, leading to the creation of an asymmetry (gradient) in sterol between these organelles. **(c)** Phosphatidyserine (PS) is produced in the ER membrane by an enzyme called Cho1. PS is then transferred by Osh6 and Osh7 to the PM by harnessing a PI4P gradient sustained by the PI 4-kinase Stt4 and Sac1, which creates an asymmetry in PS distribution (i.e. a PS gradient).

A known network is at the core of sphingolipid asymmetry in human cells. It involves enzymes that synthesize a lipid called ceramide in the ER and an enzyme that converts ceramide into sphingomyelin in the Golgi membrane. Ceramide travels from the ER to the Golgi via CERT, an LTP present in contact sites between these organelles [21]. CERT could move ceramide bidirectionally between the two membranes, but the metabolic trapping of ceramide, i.e. its conversion into sphingomyelin creates the conditions for a one-way transfer. Indeed, there is always more ceramide in the ER than in the Golgi membrane and, following the law of mass action, CERT picks up ceramide where is it abundant to release it where it is scarce (Figure 2a).

Other networks that generate asymmetry involve LTPs with a lipid exchange activity (ORPs in humans, Osh proteins in yeast). They have different molecular configurations, but all comprise a domain called ORD (OSBP-related domain) with a lipid-binding pocket [22]. Biochemical studies have revealed that Osh4, a representative member of this protein family, consisting only of an ORD, could host either a molecule of ergosterol [23] (i.e. main yeast sterol) or PI4P [24]. This enables Osh4 to exchange these ligands between two membranes [24,25]. Like other phosphoinositides, the intracellular distribution of PI4P is programmed by the organelle-specific localization of enzymes involved in its synthesis and degradation [16,17]. In the trans-Golgi membrane and the PM, PI 4-kinases add a phosphate group to PI to form PI4P, whereas in the ER the Sac1 protein hydrolyzes the bulk of PI4P into PI. Thus PI4P is mostly present in the trans-Golgi and PM, where it serves as a molecular signpost, but another consequence is that PI4P concentration gradients are created between these compartments and the ER. Data obtained with Osh4 have suggested a scenario in which this PI4P asymmetry is used by this protein to generate a sterol asymmetry [24].

In this model, Osh4 extracts a sterol molecule from the ER membrane in which the lipid is synthesized. Then it exchanges sterol for PI4P at the trans-Golgi, carries PI4P to the ER and takes another molecule of sterol. The maintenance of a PI4P gradient by the Golgi PI 4-kinase and Sac1 allows for multiple exchange cycles and the build-up of sterol in the Golgi membrane (Figure 2b). *In vitro* assays have supported this idea, first by showing that Osh4 transfers sterol and PI4P along opposite routes at maximal speed when these lipid ligands are in two distinct membranes; this is because Osh4 preferentially unloads a ligand in a membrane in which the second ligand is present. Perhaps more importantly, this has revealed that Osh4 could generate a sterol gradient between these membranes by dissipating a PI4P gradient [25]. Subsequent cellular studies on OSBP, the founding member of the ORP/Osh family, have endorsed this model, showing that OSBP plays a substantial role in building asymmetric sterol distribution by sterol/PI4P exchange [26,27]. Observations that the absence of Sac1 results in the accumulation of PI4P in the ER have further supported this model [28].

Startlingly, a similar exchange process contributes to PS asymmetry in cells. Like sterol, PS originates from the ER [15] and concentrates in the PM. How this was accomplished was hardly known [13] until the finding that Osh6 and Osh7 were PS/PI4P exchangers [29,30]. In yeast cells, these proteins harness the asymmetry in PI4P at the ER/PM interface to supply the PM with PS (Figure 2c). In human cells, ORP5 and ORP8 perform the same function [31]. The fact that PI4P requires energy to be synthesized (i.e. ATP) and is consumed in exchange cycles has led us to consider PI4P as a universal ‘fuel' used by ORP/Osh proteins to create diverse lipid asymmetries throughout the eukaryotic cells [32].

Thus, the combination of lipid transfer with lipid conversion events allows establishing one-way fluxes of lipids between membranes and create lipid asymmetries. Besides this, the features of organelle membranes likely play significant roles. The ER membrane is fluid and mostly organized into tubules, and is, therefore, highly curved [33]. These characteristics might augment the propensity of lipids to leave the ER membrane and be extracted by LTPs, as suggested *in vitro* for sterol and phospholipids [25,34,35]. Also, interestingly, some LTPs specifically localize on highly curved areas of the ER membrane [36,37]. In contrast, in the trans-Golgi membrane and the PM, the privileged association of sterol with sphingolipid presumably acts as a thermodynamic trap that prevents any sterol return to the ER [38]. Corroborating this idea, kinetic assays have shown that the delivery of sterol by sterol/PI4P exchange is enhanced in sphingolipid-rich membranes [25]. It is not clear whether sterol, which can associate laterally with PS in the PM [39,40], promotes the enrichment of this membrane with PS supplied by an ORP/Osh-mediated exchange [41].

## Why are LTPs located at membrane contact sites?

Many LTPs involved in lipid asymmetries are in contact sites for reasons that remain enigmatic. Possibly, the narrow gap between organelles allows for optimal transfer speed, but this idea is disputed and has rarely been addressed experimentally [42]. More likely, this LTP presence at contact sites guarantees local coupling between processes that make/modify lipids and those that transfer them, and, obviously, a high degree of accuracy, as lipids cannot be delivered *by mistake* to another organelle. Quite certainly, this serves to establish mechanisms that regulate lipid fluxes. The major one is a negative feedback loop encoded in the structure of many LTPs, that controls how long these proteins stay in contact sites and transfer lipids. First evidence has been provided by studies of OSBP in ER–Golgi contacts.

**Figure 3.**
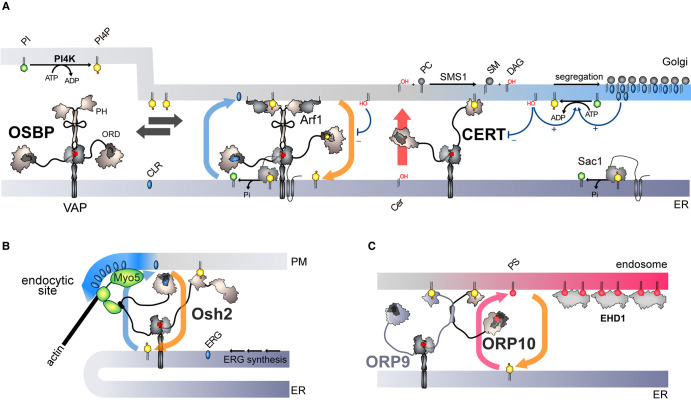
Vectorial lipid transfer at membrane contact sites. (**a**) OSBP binds to PI4P and the small G protein Arf1 localized at the surface of the Golgi membrane via its PH domain and to the VAP receptors via its FFAT motif. OSBP can thus form ER–Golgi contact sites and deliver cholesterol (CLR) in the Golgi membrane by sterol/PI4P exchange, but also promote the recruitment of CERT. This one delivers ceramide to the trans-Golgi, which leads to the synthesis of SM and DAG. The accumulation of DAG and sterol enhances PI4P synthesis through diverse mechanisms. DAG negatively regulates the association of OSBP and CERT with the Golgi membrane. The consumption of PI4P and the production of DAG eventually trigger the disassembly of ER–Golgi contacts, which stops both sterol transfer and SM production. (**b**) Osh2 is in ER–PM contact sites in association with Myo5, and delivers ergosterol at endocytic sites by sterol/PI4P exchange. (**c**) ORP10 is in ER-endosome contact sites and delivers PS to the endosomal membrane by PS/PI4P exchange, which promotes the recruitment of EHD1 protein. Intriguingly, ORP10 is attached to the ER by forming a heterodimer with the sterol-binding protein ORP9 in complex with VAP. The functional meaning of such a partnership is not completely understood.

OSBP is a sterol/PI4P exchanger more complex than Osh4 that can connect the ER with the trans-Golgi membrane in addition to transferring lipids (Figure 3a); it comprises a short amino-acid motif to bind with the ER-resident protein VAPs [43] and a structural domain called PH (Pleckstrin Homology) to dock onto the trans-Golgi by recognizing PI4P [44]. It is surprising that OSBP depends on PI4P to associate with the Golgi membrane while having the ability to extract and transfer this lipid. This is in fact critical to implement a negative feedback loop that controls lipid transfer at ER–Golgi contacts [26]. As long as PI4P is available in the Golgi membrane, OSBP forms ER–Golgi contacts and exchanges PI4P for sterol, leading to the accumulation of cholesterol in the Golgi membrane. Once PI4P is exhausted, due to its transfer to the ER and hydrolysis by Sac1, OSBP dissociates from the Golgi membrane, exits contact sites and becomes inactive.

Moreover, via its exchange activity, OSBP controls the transfer activity of CERT, which also depends on PI4P to populate ER–Golgi contacts and be operative [45]. Consequently, the ceramide and cholesterol flows are synchronized, allowing for a strict control of the sterol/sphingolipid balance in the Golgi apparatus. Additional processes tightly regulate these flows. Notably, the accumulation in the Golgi membrane of sterol and DAG, a by-product of sphingomyelin synthesis, modulates both the synthesis of PI4P and the activities of CERT and OSBP [27,45–51]. OSBP must be spatially close to PI 4-kinases; otherwise lipid exchange is decoupled from PI4P synthesis, which results in uncontrolled PI4P waves along the Golgi apparatus and aberrant dynamics in ER–Golgi contacts formation [27]. Also, Sac1 associates with OSBP in complex with VAP [52]. Thus, in ER–Golgi contacts, OSBP and CERT are confined together with enzymes of the PI4P metabolism and auxiliary proteins to determine the lipid composition of the Golgi apparatus. This is critical for the formation of transport vesicles that carry proteins from the Golgi to the PM [52] and has an impact on the inner organization of the PM [53].

Finally, as suggested by these data, a reason why LTPs are secluded in contact sites is probably to combine their activities with those of local protein partners, and thereby perform specialized cellular functions. Recent studies have confirmed this notion, showing that diverse ORP/Osh proteins exchange lipids at contact sites to support specific vesicular trafficking routes. In yeast, Osh2 functions as a sterol/PI4P exchanger in ER–PM contacts, where it associates with Myo5, a protein required for actin polymerization and scission of endocytic vesicles (Figure 3b) [54]. Myo5 also interacts with ER-resident proteins involved in ergosterol biosynthesis [55]. Presumably, all these proteins co-operate to channel ergosterol to the PM and create sterol-rich membrane domains where actin polymerization is facilitated to promote endocytosis [55]. In contrast, it is likely that Osh1, which occupies ER–Golgi contacts, performs sterol/PI4P exchange to regulate post-Golgi vesicular trafficking [56]. In human cells, ORP10 exchanges PS for PI4P in ER-endosome contacts to deliver PS to the endosomal membrane (Figure 3c). This promotes the recruitment of the EHD1 protein whose membrane scission activity triggers the formation of tubulovesicular carriers that recycle proteins from the endosomes to the Golgi [57].

## Massive lipid transfer or fine-tuning of local lipid content?

If today we are aware of the existence of protein networks that ensure one-way lipid transfers, it remains difficult to quantify how much they contribute to the impressive asymmetries in lipid distribution observed in the cell. First, although many approaches have been developed to map cellular lipid routes, for instance by using fluorescent lipids [58], lipid labelling procedures [58] or genetically encoded lipid sensors [58,59], these methods are not entirely suitable for precisely quantifying lipid fluxes. Second, *in vitro* assays provide key insights into the function of LTPs, but they are not realistic enough to define the exact speed at which these proteins carry lipids in the complex environment of the cell [60].

Attempts have been made to estimate how much lipid is transferred by given LTPs in cells considering their abundance and the speed at which they transfer their lipid ligands *in vitro* [25,60]. Calculations have also been made in specific cases in which the cellular abundance and contribution of LTPs to the synthesis of particular lipid species via the transport of their precursor were known [60]. Regarding ORP/Osh activity, insights came from the analysis of cellular PI4P levels. This lipid is present in minute amounts in the cell (in yeast, only 0.2% of lipids [61]) compared with sterol and PS, which seems incompatible with ORP/Osh-mediated exchange processes. Yet, in yeast lacking Sac1 or all Osh proteins, PI4P levels can be multiplied by up to ∼20 [62,63]. This suggests that substantial quantities of PI4P are constantly produced and consumed by networks involving Sac1 and ORP/Osh proteins to move lipids. In parallel, Osh6 and Osh7 were found to be responsible for 30% of PS delivery to the PM [29]. Later, investigations revealed that OSBP uses half of PI4P synthesized in a human cell to mediate 30–60% of ER-to-Golgi sterol transfer [27]. Thus ORP/Osh proteins seem able to transfer significant amount of lipids for creating lipid asymmetries.

However, in different cases, the *raison d’être* of ORP/Osh proteins is perhaps not to use the PI4P metabolism to deliver massive quantities of lipids to cellular membranes. Osh4 served as a great prototypical case to uncover PI4P-driven lipid exchange, but paradoxically there is no direct evidence besides a few clues that it delivers sterol in the Golgi in yeast cells [5,64]. Instead, it seems more certain that its role is to lower PI4P levels in post-Golgi secretory vesicles for functional purposes [65,66]. Also, OSBP was found to localize at ER-endosome contacts to control protein recycling not by delivering sterol but by adjusting the level of a small pool of endosomal PI4P [67]. Possibly, these proteins perform limited sterol/PI4P exchange to regulate the PI4P level of given membranes or control their sterol/PI4P balance. Another idea is that a LTP like Osh4 acts as a negative regulator of PI4P signalling by sequestering this lipid, and that a high concentration of sterol lifts its inhibitory action [68].

**Figure 4.**
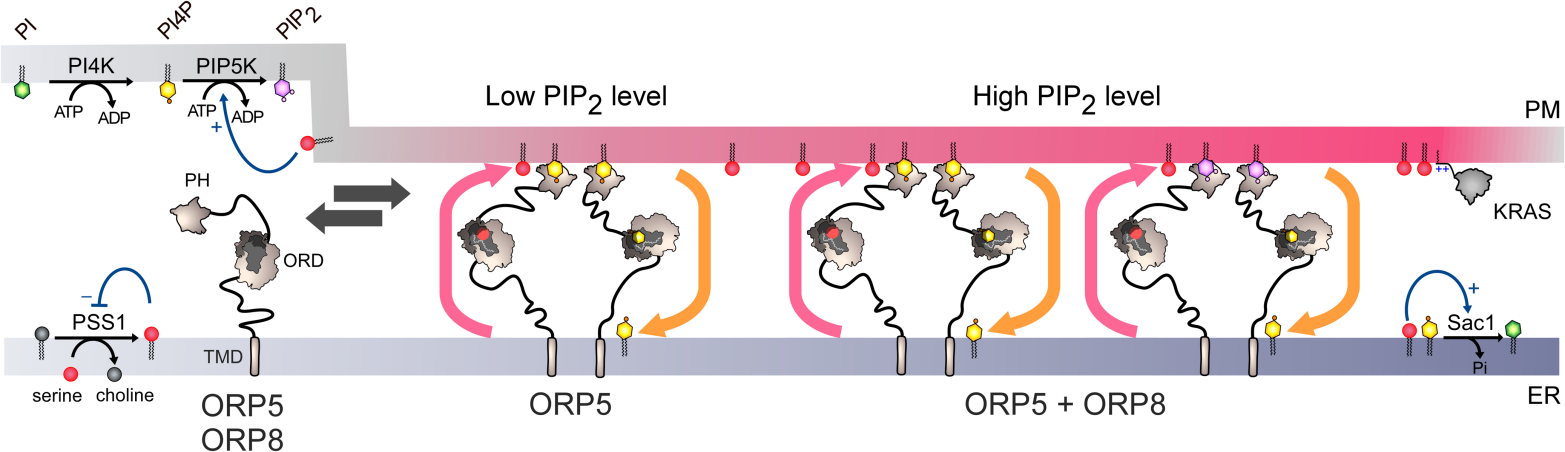
A rheostat mechanism to control PS, PI4P and PIP_2_ levels in the plasma membrane. ORP5 and ORP8 are anchored to the ER by a transmembrane domain (TMD) and associate with the PM by interacting with PI4P and/or PIP_2_ via their PH domain to join ER–PM contacts. When the PIP_2_ levels are high, ORP8 is recruited together with ORP5, and both proteins supply PS while decreasing the PI4P level, which limits PIP_2_ synthesis. At a low PIP_2_ level, only ORP5 docks and supplies PS to the PM unless the PI4P pool is exhausted. When there is not enough PI4P and PIP_2_, ORP5 and ORP8 disengage from contact sites. PSS1 synthesizes PS and its activity is inhibited by the end-product of the reaction. PS likely activates Sac1 in the ER and promotes the synthesis of PIP_2_ in the PM. This complex protein network determines the lipid content of the PM, which impacts the recruitment of the oncogenic protein KRAS by electrostatic interaction.

Moreover, studies of ORP5 and ORP8 in humans have shown that besides supplying the PM with PS, they also regulate the level of the main signalling lipid present in this membrane, which is PIP_2_. These proteins are ER-anchored [69,70] and associate with the PM if the latter contains threshold PI4P and PIP_2_ amounts, to join ER–PM contacts [31,71,72]. The PM is then enriched in PS but its content in PIP_2_ decreases [71,72]. This is because PI4P, which is used as fuel for PS/PI4P exchange also serves as substrate for PIP_2_ synthesis (it is unlikely that PS and PIP_2_ are directly exchanged [41,72]). When PI4P and PIP_2_ levels become too low, the proteins exit contact sites, PS transfer stops, and proper phosphoinositide levels are restored. So, ORP5 and ORP8 seem to constitute the molecular basis of a complex mechanism whose function is more to finely adjust the lipid composition of the PM, and thereby the signalling capacity of the cells [72], than to massively import PS in the PM (Figure 4). Side mechanisms are thought to regulate PS and PI4P flows. For instance, PS inhibits its own synthesis in the ER and this strongly influences PS/PI4P exchange [73]. Sac1 can be activated by PS [74], suggesting that an elevation of PS in the ER could generate a steeper PI4P gradient to sustain lipid exchange. PS might also promote the conversion of PI4P into PIP_2_ by PI4P 5-kinase in the PM [75]. It is increasingly appreciated that dysregulation of this complex protein network can impact the recruitment of oncogenic proteins (e.g. KRAS) at the PM, and be associated with cancers [76].

## Sensing lipid asymmetry to rectify cellular lipid levels

**Figure 5.**
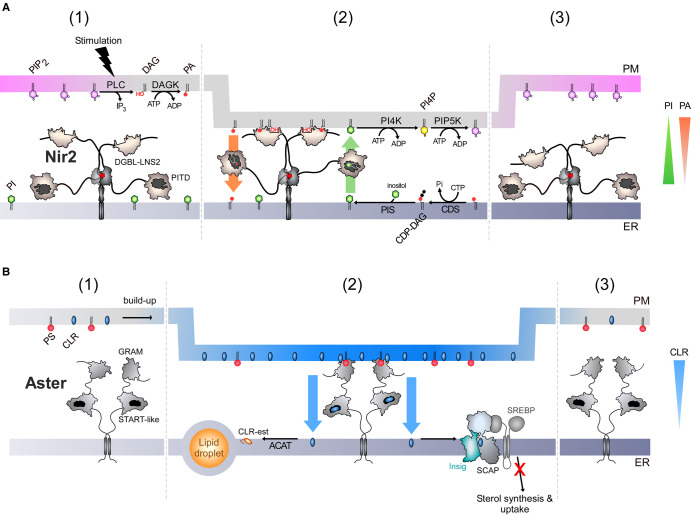
Sensing and exploiting a change in lipid asymmetry to rectify cellular lipid content. (**a**) Nir2 is associated with the ER-resident VAP receptor via its FFAT motif (step 1). Following the accumulation of DAG and PA resulting from the hydrolysis of PIP_2_, Nir2 engages the PM via its PA-binding domain LNS2 (Lipin/Nde1/Smp2) and DAG-binding-like domain (DGBL). PI is already present in the ER; thus the presence of PA in the PM creates the conditions for immediate PA/PI exchange as PI and PA are concentrated in distinct membranes. PA and PI could thus be rapidly exchanged by the PITD domain of Nir2, which can host the two lipids in a mutually exclusive manner. The conversion of PA into PI likely sustains this exchange until the full clearance of PA from the PM and the replenishment of PIP_2_ levels (step 2). Then Nir2 disengages from ER–PM contacts (step 3). PLC: phospholipase, DAGK: DAG kinase; CDS: CDP-DAG synthase, PIS: PI synthase; PI4K: PI 4-kinase; PIP5K: PI4P 5-kinase. (**b**) Aster proteins are anchored to the ER membrane and form dimeric complexes (step 1). Following a transient expansion of the accessible pool of sterol in the PM, they localize to ER–PM contacts, by detecting the concomitant presence of cholesterol and PS in the inner leaflet of the PM (step 2). Cholesterol is then transferred by the START-like domain of Aster, down its concentration gradient, to the ER, where it is esterified and stored in lipid droplets. The increase in the ER cholesterol level also leads to the down-regulation at the transcriptional level, of sterol synthesis and uptake via the SREBP2 pathway (involving Insig/SCAP/SREBP proteins). When sterol levels are rectified, Aster proteins exit ER–PM contacts (step 3). In theory, an increase in the sterol level of the inner leaflet of the PM, caused by a mixing of lipids (scrambling) of the two membrane leaflets, is unlikely to trigger the recruitment of Aster. Indeed, PS, which is mostly present in the inner leaflet would be simultaneously diluted across the membrane. CLR: cholesterol; CLR-est: Cholesteryl ester; ACAT: Acyl-CoA cholesterol acyltransferase.

Some networks involving LTPs are clearly tailored not to create lipid asymmetries but to sense a change in asymmetry and subsequently rectify the cellular level of particular lipids. One of these is critical to guarantee the signalling competence of the cell. In many signalling pathways, the activation of cell surface receptors triggers the hydrolysis of PIP_2_ by phospholipase, which generates DAG and IP_3_ as second messengers. PIP_2_ levels must subsequently be restored so that the cells can respond once again to stimulation. This requires several enzymatic steps that constitute the PI cycle [77]. First, DAG is converted into phosphatidic acid (PA) in the PM. Next, PA is converted into PI in the ER membrane. Finally, in the PM, PI undergoes two successive phosphorylations leading to PIP_2_ re-synthesis (Figure 5a). As hypothesized as early as 1975, the PI cycle can function only if PA and PI are exchanged between the ER and the PM to guarantee lipid flows between the spatially distant enzymes of this cycle [78]. As described below, long-term efforts have led to the identification of LTPs — Nir2 and Nir3 in humans — as the missing pieces of the puzzle,

These proteins are attached to the ER [79] and have a domain to alternatively capture PA and PI [80]. In addition, they possess a PA-binding domain and likely a DAG-binding domain to associate with the PM [81,82]. Recent data suggest the following scenario. The generation of DAG and PA triggers the translocation of Nir proteins to ER–PM contacts [82]. The accumulation of PA in the PM creates an asymmetry in PA at ER–PM contacts. Because PI is absent from the PM and already abundant in the ER [83,84], this likely sets the conditions for immediate PA/PI exchange as PA and PI are concentrated in distinct membranes. Next, PA is converted into PI in the ER, which sustains the exchange process until a full clearance of PA [82] and replenishment of the PIP_2_ stock. Finally, as PA is cleared from the PM, Nir proteins disengage from contact sites. Interestingly, compared with Nir2, Nir3 has a weaker PA-transfer capacity but a higher capacity to detect small amounts of PA in the PM [85]. Consequently, Nir3 adjusts the PIP_2_ level in resting cells, whereas Nir2 is only mobilized during acute receptor activation.

A second example was provided by the characterization of a newly discovered family of LTPs (Asters in humans, Lam/Ltc in yeast). These are ER-anchored proteins that have a PH-like domain called GRAM to associate with a second organelle and a sterol-transfer module [86,87]. Studies have shown that these proteins occupy contact sites between the ER and the mitochondrion, vacuole or PM to potentially serve as sterol transporters [86,87]. Remarkably, the GRAM domain of Asters (also called GRAMD1) has two binding sites for recognizing cholesterol and PS in a synergistic manner [88–90]. Consequently, in human cells, Asters can specifically join ER–PM contacts by sensing in the PM, in which PS is present, the transient expansion of an accessible pool of cholesterol resulting from the uptake of sterol by endocytosis of low-density or high-density lipoproteins [88,90–92]. Next, Asters transfer cholesterol down its concentration gradient to the ER; cholesterol is subsequently esterified to be stored in lipid droplets [91,92] (Figure 5b). Additionally, the sterol level increase in the ER down-regulates via the SREBP2 pathways the transcription of gene coding for the synthesis of cholesterol and its uptake [88,89,91,92]. Remarkably, Asters use their transfer activity to act as messengers. They inform the regulatory mechanisms that control the biosynthesis and uptake of sterol, present in the ER, of the overabundance of cholesterol in the PM, preventing the toxic accumulation of this lipid in the cell. This has important implications, since defects in this mechanism can result in a lack of steroid hormone synthesis in model animals [91] and might be linked to intellectual disability in humans [89].

## Conclusion and perspectives

LTPs assisted by lipid-synthesizing enzymes vectorially transfer lipids between organelles. It is critical to better quantify whether this serves to create lipid asymmetries throughout the cell and/or to fine-tune the lipid content of organelles. More work is also required to better define which lipid ligands are recognized by different LTPs and/or whether these LTPs function as lipid exchangers or mere lipid transporters. In some cases, the mechanism by which an LTP accumulates lipids in organelles remains particularly elusive [93]. In parallel, significant efforts are being undertaken to dissect networks present at ER-mitochondrion contacts and inside the mitochondria that contribute to the generation of key cellular lipids, among which specific mitochondrial ones [94]. The recent discovery of diverse LTPs at the mitochondrial surface raises exciting questions [95,96]. Finally, some evidence suggests that vesicular trafficking also contributes to creating lipid asymmetry via lipid sorting mechanisms whose characteristics remain to be defined [5,97,98]. In the future, a better understanding of how asymmetries in lipid distribution are created should provide key outcomes in basic cell biology and answer health-related questions.

## Summary

- LTPs assisted by lipid-synthesizing enzymes can move lipids in a directional manner between two cellular membranes.
- Lipid transfers mostly occur in contact sites where membranes are extremely close for several functional purposes.
- These transfer processes contribute to creating asymmetric lipid distributions in the cell and/or fine-tuning the lipid composition of cellular membranes.
- Some LTPs play a key role in mechanisms that rectify the cellular level of particular lipids following changes in lipid asymmetry.

## Abbreviations

CERT: ceramide transfer protein
DAG: diacylglycerol
ER: endoplasmic reticulum
LTP: lipid transfer protein
ORD: OSBP-related domain
ORP: oxysterol-binding protein related-proteins
Osh: oxysterol-binding homology
PA: phosphatidic acid
PH: Pleckstrin Homology
PI: phosphatidylinositol
PI4P: phosphatidylinositol 4-phosphate
PIP_2_: phosphatidylinositol 4,5-bisphosphate
PM: plasma membrane

## References

[1] Holthuis, J.C. and Menon, A.K. (2014) Lipid landscapes and pipelines in membrane homeostasis. Nature 510, 48–57 10.1038/nature13474 24899304

[2] Bigay, J. and Antonny, B. (2012) Curvature, lipid packing, and electrostatics of membrane organelles: defining cellular territories in determining specificity. Dev. Cell 23, 886–895 10.1016/j.devcel.2012.10.009 23153485

[3] Ejsing, C.S., Sampaio, J.L., Surendranath, V., Duchoslav, E., Ekroos, K., Klemm, R.W. et al. (2009) Global analysis of the yeast lipidome by quantitative shotgun mass spectrometry. Proc. Natl Acad. Sci. U.S.A. 106, 2136–2141 10.1073/pnas.0811700106 19174513 PMC2650121

[4] Sampaio, J.L., Gerl, M.J., Klose, C., Ejsing, C.S., Beug, H., Simons, K. et al. (2011) Membrane lipidome of an epithelial cell line. Proc. Natl Acad. Sci. U.S.A. 108, 1903–1907 10.1073/pnas.1019267108 21245337 PMC3033259

[5] Klemm, R.W., Ejsing, C.S., Surma, M.A., Kaiser, H.J., Gerl, M.J., Sampaio, J.L. et al. (2009) Segregation of sphingolipids and sterols during formation of secretory vesicles at the trans-Golgi network. J. Cell Biol. 185, 601–612 10.1083/jcb.200901145 19433450 PMC2711577

[6] Zinser, E., Sperka-Gottlieb, C.D., Fasch, E.V., Kohlwein, S.D., Paltauf, F. and Daum, G. (1991) Phospholipid synthesis and lipid composition of subcellular membranes in the unicellular eukaryote *Saccharomyces cerevisiae*. J. Bacteriol. 173, 2026–2034 10.1128/jb.173.6.2026-2034.1991 2002005 PMC207737

[7] Schneiter, R., Brugger, B., Sandhoff, R., Zellnig, G., Leber, A., Lampl, M. et al. (1999) Electrospray ionization tandem mass spectrometry (ESI-MS/MS) analysis of the lipid molecular species composition of yeast subcellular membranes reveals acyl chain-based sorting/remodeling of distinct molecular species en route to the plasma membrane. J. Cell Biol. 146, 741–754 10.1083/jcb.146.4.741 10459010 PMC2156145

[8] Andreyev, A.Y., Fahy, E., Guan, Z., Kelly, S., Li, X., McDonald, J.G. et al. (2010) Subcellular organelle lipidomics in TLR-4-activated macrophages. J. Lipid Res. 51, 2785–2797 10.1194/jlr.M008748 20574076 PMC2918461

[9] Mesmin, B. and Maxfield, F.R. (2009) Intracellular sterol dynamics. Biochim. Biophys. Acta 1791, 636–645 10.1016/j.bbalip.2009.03.002 19286471 PMC2696574

[10] Levental, I., Levental, K.R. and Heberle, F.A. (2020) Lipid rafts: controversies resolved, mysteries remain. Trends Cell Biol. 30, 341–353 10.1016/j.tcb.2020.01.009 32302547 PMC7798360

[11] Daum, G., Tuller, G., Nemec, T., Hrastnik, C., Balliano, G., Cattel, L. et al. (1999) Systematic analysis of yeast strains with possible defects in lipid metabolism. Yeast 15, 601–614 10.1002/(SICI)1097-0061(199905)15:7&lt;601::AID-YEA390>3.0.CO;2-N 10341423

[12] Vance, J.E. and Steenbergen, R. (2005) Metabolism and functions of phosphatidylserine. Prog. Lipid Res. 44, 207–234 10.1016/j.plipres.2005.05.001 15979148

[13] Leventis, P.A. and Grinstein, S. (2010) The distribution and function of phosphatidylserine in cellular membranes. Annu. Rev. Biophys. 39, 407–427 10.1146/annurev.biophys.093008.131234 20192774

[14] Kay, J.G. and Fairn, G.D. (2019) Distribution, dynamics and functional roles of phosphatidylserine within the cell. Cell Commun. Signal. 17, 126 10.1186/s12964-019-0438-z 31615534 PMC6792266

[15] Lenoir, G., D'Ambrosio, J.M., Dieudonne, T. and Copic, A. (2021) Transport pathways that contribute to the cellular distribution of phosphatidylserine. Front. Cell Dev. Biol. 9, 737907 10.3389/fcell.2021.737907 34540851 PMC8440936

[16] Posor, Y., Jang, W. and Haucke, V. (2022) Phosphoinositides as membrane organizers. Nat. Rev. Mol. Cell Biol. 10.1038/s41580-022-00490-x PMC9117997 35589852

[17] Dickson, E.J. and Hille, B. (2019) Understanding phosphoinositides: rare, dynamic, and essential membrane phospholipids. Biochem. J. 476, 1–23 10.1042/BCJ20180022 30617162 PMC6342281

[18] Wong, L.H., Gatta, A.T. and Levine, T.P. (2019) Lipid transfer proteins: the lipid commute via shuttles, bridges and tubes. Nat. Rev. Mol. Cell Biol. 20, 85–101 10.1038/s41580-018-0071-5 30337668

[19] Egea, P.F. (2021) Mechanisms of non-vesicular exchange of lipids at membrane contact sites: of shuttles, tunnels and, funnels. Front. Cell Dev. Biol. 9, 784367 10.3389/fcell.2021.784367 34912813 PMC8667587

[20] Scorrano, L., De Matteis, M.A., Emr, S., Giordano, F., Hajnoczky, G., Kornmann, B. et al. (2019) Coming together to define membrane contact sites. Nat. Commun. 10, 1287 10.1038/s41467-019-09253-3 30894536 PMC6427007

[21] Hanada, K., Kumagai, K., Yasuda, S., Miura, Y., Kawano, M., Fukasawa, M. et al. (2003) Molecular machinery for non-vesicular trafficking of ceramide. Nature 426, 803–809 10.1038/nature02188 14685229

[22] Delfosse, V., Bourguet, W. and Drin, G. (2020) Structural and functional specialization of OSBP-related proteins. Contact 3, 2515256420946627 10.1177/2515256420946627

[23] Im, Y.J., Raychaudhuri, S., Prinz, W.A. and Hurley, J.H. (2005) Structural mechanism for sterol sensing and transport by OSBP-related proteins. Nature 437, 154–158 10.1038/nature03923 16136145 PMC1431608

[24] de Saint-Jean, M., Delfosse, V., Douguet, D., Chicanne, G., Payrastre, B., Bourguet, W. et al. (2011) Osh4p exchanges sterols for phosphatidylinositol 4-phosphate between lipid bilayers. J. Cell Biol. 195, 965–978 10.1083/jcb.201104062 22162133 PMC3241724

[25] Moser von Filseck, J., Vanni, S., Mesmin, B., Antonny, B. and Drin, G. (2015) A phosphatidylinositol-4-phosphate powered exchange mechanism to create a lipid gradient between membranes. Nat. Commun. 6, 6671 10.1038/ncomms7671 25849868

[26] Mesmin, B., Bigay, J., von Filseck J, M., Lacas-Gervais, S., Drin, G. and Antonny, B. (2013) A four-step cycle driven by PI(4)P hydrolysis directs sterol/PI(4)P exchange by the ER-Golgi tether OSBP. Cell 155, 830–843 10.1016/j.cell.2013.09.056 24209621

[27] Mesmin, B., Bigay, J., Polidori, J., Jamecna, D., Lacas-Gervais, S. and Antonny, B. (2017) Sterol transfer, PI4P consumption, and control of membrane lipid order by endogenous OSBP. EMBO J. 36, 3156–3174 10.15252/embj.201796687 28978670 PMC5666618

[28] Zewe, J.P., Wills, R.C., Sangappa, S., Goulden, B.D. and Hammond, G.R. (2018) SAC1 degrades its lipid substrate PtdIns4P in the endoplasmic reticulum to maintain a steep chemical gradient with donor membranes. eLife 7, e35588 10.7554/eLife.35588 29461204 PMC5829913

[29] Maeda, K., Anand, K., Chiapparino, A., Kumar, A., Poletto, M., Kaksonen, M. et al. (2013) Interactome map uncovers phosphatidylserine transport by oxysterol-binding proteins. Nature 501, 257–261 10.1038/nature12430 23934110

[30] Moser von Filseck, J., Copic, A., Delfosse, V., Vanni, S., Jackson, C.L., Bourguet, W. et al. (2015) INTRACELLULAR TRANSPORT. Phosphatidylserine transport by ORP/Osh proteins is driven by phosphatidylinositol 4-phosphate. Science 349, 432–436 10.1126/science.aab1346 26206936

[31] Chung, J., Torta, F., Masai, K., Lucast, L., Czapla, H., Tanner, L.B. et al. (2015) INTRACELLULAR TRANSPORT. PI4P/phosphatidylserine countertransport at ORP5- and ORP8-mediated ER-plasma membrane contacts. Science 349, 428–432 10.1126/science.aab1370 26206935 PMC4638224

[32] Moser von Filseck, J. and Drin, G. (2016) Running up that hill: how to create cellular lipid gradients by lipid counter-flows. Biochimie 130, 115–121 10.1016/j.biochi.2016.08.001 27519300

[33] West, M., Zurek, N., Hoenger, A. and Voeltz, G.K. (2011) A 3D analysis of yeast ER structure reveals how ER domains are organized by membrane curvature. J. Cell Biol. 193, 333–346 10.1083/jcb.201011039 21502358 PMC3080256

[34] Thomas, P.D. and Poznansky, M.J. (1988) Effect of surface curvature on the rate of cholesterol transfer between lipid vesicles. Biochem. J. 254, 155–160 10.1042/bj2540155 3178745 PMC1135051

[35] Sugiura, T., Nakao, H., Ikeda, K., Khan, D., Nile, A.H., Bankaitis, V.A. et al. (2021) Biophysical parameters of the Sec14 phospholipid exchange cycle - effect of lipid packing in membranes. Biochim. Biophys. Acta Biomembr. 1863, 183450 10.1016/j.bbamem.2020.183450 32828847 PMC7982300

[36] Hoffmann, P.C., Bharat, T.A.M., Wozny, M.R., Boulanger, J., Miller, E.A. and Kukulski, W. (2019) Tricalbins contribute to cellular lipid flux and form curved ER-PM contacts that are bridged by rod-shaped structures. Dev. Cell 51, 488–502.e8 10.1016/j.devcel.2019.09.019 31743663 PMC6863393

[37] Collado, J., Kalemanov, M., Campelo, F., Bourgoint, C., Thomas, F., Loewith, R. et al. (2019) Tricalbin-mediated contact sites control ER curvature to maintain plasma membrane integrity. Dev. Cell 51, 476–487.e7 10.1016/j.devcel.2019.10.018 31743662 PMC6863395

[38] Baumann, N.A., Sullivan, D.P., Ohvo-Rekila, H., Simonot, C., Pottekat, A., Klaassen, Z. et al. (2005) Transport of newly synthesized sterol to the sterol-enriched plasma membrane occurs via nonvesicular equilibration. Biochemistry 44, 5816–5826 10.1021/bi048296z 15823040

[39] Hirama, T., Lu, S.M., Kay, J.G., Maekawa, M., Kozlov, M.M., Grinstein, S. et al. (2017) Membrane curvature induced by proximity of anionic phospholipids can initiate endocytosis. Nat. Commun. 8, 1393 10.1038/s41467-017-01554-9 29123120 PMC5680216

[40] Maekawa, M. and Fairn, G.D. (2015) Complementary probes reveal that phosphatidylserine is required for the proper transbilayer distribution of cholesterol. J. Cell Sci. 128, 1422 10.1242/jcs.164715 25663704

[41] Ikhlef, S., Lipp, N.-F., Delfosse, V., Fuggetta, N., Bourguet, W., Magdeleine, M. et al. (2021) Functional analyses of phosphatidylserine/PI(4)P exchangers with diverse lipid species and membrane contexts reveal unanticipated rules on lipid transfer. BMC Biol. 19, 248 10.1186/s12915-021-01183-1 34801011 PMC8606082

[42] Dittman, J.S. and Menon, A.K. (2017) Speed limits for nonvesicular intracellular sterol transport. Trends Biochem. Sci. 42, 90–97 10.1016/j.tibs.2016.11.004 27956059 PMC5272819

[43] Loewen, C.J., Roy, A. and Levine, T.P. (2003) A conserved ER targeting motif in three families of lipid binding proteins and in Opi1p binds VAP. EMBO J. 22, 2025–2035 10.1093/emboj/cdg201 12727870 PMC156073

[44] Levine, T.P. and Munro, S. (2002) Targeting of Golgi-specific pleckstrin homology domains involves both PtdIns 4-kinase-dependent and -independent components. Curr. Biol. 12, 695–704 10.1016/S0960-9822(02)00779-0 12007412

[45] Capasso, S., Sticco, L., Rizzo, R., Pirozzi, M., Russo, D., Dathan, N.A. et al. (2017) Sphingolipid metabolic flow controls phosphoinositide turnover at the trans-Golgi network. EMBO J. 36, 1736–1754 10.15252/embj.201696048 28495678 PMC5470045

[46] Prashek, J., Bouyain, S., Fu, M., Li, Y., Berkes, D. and Yao, X. (2017) Interaction between the PH and START domains of ceramide transfer protein competes with phosphatidylinositol 4-phosphate binding by the PH domain. J. Biol. Chem. 292, 14217–14228 10.1074/jbc.M117.780007 28652409 PMC5572904

[47] Sugiki, T., Egawa, D., Kumagai, K., Kojima, C., Fujiwara, T., Takeuchi, K. et al. (2018) Phosphoinositide binding by the PH domain in ceramide transfer protein (CERT) is inhibited by hyperphosphorylation of an adjacent serine-repeat motif. J. Biol. Chem. 293, 11206–11217 10.1074/jbc.RA118.002465 29848549 PMC6052210

[48] Hausser, A., Storz, P., Martens, S., Link, G., Toker, A. and Pfizenmaier, K. (2005) Protein kinase D regulates vesicular transport by phosphorylating and activating phosphatidylinositol-4 kinase IIIbeta at the Golgi complex. Nat. Cell Biol. 7, 880–886 10.1038/ncb1289 16100512 PMC1458033

[49] Nhek, S., Ngo, M., Yang, X., Ng, M.M., Field, S.J., Asara, J.M. et al. (2010) Regulation of oxysterol-binding protein Golgi localization through protein kinase D-mediated phosphorylation. Mol. Biol. Cell 21, 2327–2337 10.1091/mbc.E10-02-0090 20444975 PMC2893995

[50] Fugmann, T., Hausser, A., Schoffler, P., Schmid, S., Pfizenmaier, K. and Olayioye, M.A. (2007) Regulation of secretory transport by protein kinase D-mediated phosphorylation of the ceramide transfer protein. J. Cell Biol. 178, 15–22 10.1083/jcb.200612017 17591919 PMC2064413

[51] Lu, D., Sun, H.Q., Wang, H., Barylko, B., Fukata, Y., Fukata, M. et al. (2012) Phosphatidylinositol 4-kinase IIalpha is palmitoylated by Golgi-localized palmitoyltransferases in cholesterol-dependent manner. J. Biol. Chem. 287, 21856–21865 10.1074/jbc.M112.348094 22535966 PMC3381148

[52] Wakana, Y., Kotake, R., Oyama, N., Murate, M., Kobayashi, T., Arasaki, K. et al. (2015) CARTS biogenesis requires VAP-lipid transfer protein complexes functioning at the endoplasmic reticulum-Golgi interface. Mol. Biol. Cell 26, 4686–4699 10.1091/mbc.E15-08-0599 26490117 PMC4678024

[53] Malek, M., Wawrzyniak, A.M., Koch, P., Luchtenborg, C., Hessenberger, M., Sachsenheimer, T. et al. (2021) Inositol triphosphate-triggered calcium release blocks lipid exchange at endoplasmic reticulum-Golgi contact sites. Nat. Commun. 12, 2673 10.1038/s41467-021-22882-x 33976123 PMC8113574

[54] Encinar del Dedo, J., Idrissi, F.Z., Fernandez-Golbano, I.M., Garcia, P., Rebollo, E., Krzyzanowski, M.K. et al. (2017) ORP-mediated ER contact with endocytic sites facilitates actin polymerization. Dev. Cell 43, 588–602.e6 10.1016/j.devcel.2017.10.031 29173820

[55] Encinar del Dedo, J., Fernandez-Golbano, I.M., Pastor, L., Meler, P., Ferrer-Orta, C., Rebollo, E. et al. (2021) Coupled sterol synthesis and transport machineries at ER-endocytic contact sites. J. Cell Biol. 220, e202010016 10.1083/jcb.202010016 34283201 PMC8294947

[56] Shin, J.J.H., Liu, P., Chan, L.J., Ullah, A., Pan, J., Borchers, C.H. et al. (2020) Ph biosensing by PI4P regulates cargo sorting at the TGN. Dev. Cell 52, 461–476.e4 10.1016/j.devcel.2019.12.010 31928972

[57] Kawasaki, A., Sakai, A., Nakanishi, H., Hasegawa, J., Taguchi, T., Sasaki, J. et al. (2022) PI4P/PS countertransport by ORP10 at ER-endosome membrane contact sites regulates endosome fission. J. Cell Biol. 221, e202103141 10.1083/jcb.202103141 34817532 PMC8624802

[58] Schoop, V., Martello, A., Eden, E.R. and Hoglinger, D. (2021) Cellular cholesterol and how to find it. Biochim. Biophys. Acta Mol. Cell. Biol. Lipids 1866, 158989 10.1016/j.bbalip.2021.158989 34118431

[59] Hammond, G.R.V., Ricci, M.M.C., Weckerly, C.C. and Wills, R.C. (2022) An update on genetically encoded lipid biosensors. Mol. Biol. Cell 33, tp2 10.1091/mbc.E21-07-0363 35420888 PMC9282013

[60] Wong, L.H., Copic, A. and Levine, T.P. (2017) Advances on the transfer of lipids by lipid transfer proteins. Trends Biochem. Sci. 42, 516–530 10.1016/j.tibs.2017.05.001 28579073 PMC5486777

[61] Audhya, A., Foti, M. and Emr, S.D. (2000) Distinct roles for the yeast phosphatidylinositol 4-kinases, Stt4p and Pik1p, in secretion, cell growth, and organelle membrane dynamics. Mol. Biol. Cell 11, 2673–2689 10.1091/mbc.11.8.2673 10930462 PMC14948

[62] Foti, M., Audhya, A. and Emr, S.D. (2001) Sac1 lipid phosphatase and Stt4 phosphatidylinositol 4-kinase regulate a pool of phosphatidylinositol 4-phosphate that functions in the control of the actin cytoskeleton and vacuole morphology. Mol. Biol. Cell 12, 2396–2411 10.1091/mbc.12.8.2396 11514624 PMC58602

[63] Stefan, C.J., Manford, A.G., Baird, D., Yamada-Hanff, J., Mao, Y. and Emr, S.D. (2011) Osh proteins regulate phosphoinositide metabolism at ER-plasma membrane contact sites. Cell 144, 389–401 10.1016/j.cell.2010.12.034 21295699

[64] Fairn, G.D., Curwin, A.J., Stefan, C.J. and McMaster, C.R. (2007) The oxysterol binding protein Kes1p regulates Golgi apparatus phosphatidylinositol-4-phosphate function. Proc. Natl Acad. Sci. U.S.A. 104, 15352–15357 10.1073/pnas.0705571104 17881569 PMC2000554

[65] Smindak, R.J., Heckle, L.A., Chittari, S.S., Hand, M.A., Hyatt, D.M., Mantus, G.E. et al. (2017) Lipid-dependent regulation of exocytosis in *S. cerevisiae* by OSBP homolog (Osh) 4. J. Cell Sci. 130, 3891–3906 10.1242/jcs.205435 28993464

[66] Ling, Y., Hayano, S. and Novick, P. (2014) Osh4p is needed to reduce the level of phosphatidylinositol-4-phosphate on secretory vesicles as they mature. Mol. Biol. Cell 25, 3389–3400 10.1091/mbc.E14-06-1087 25165144 PMC4214785

[67] Dong, R., Saheki, Y., Swarup, S., Lucast, L., Harper, J.W. and De Camilli, P. (2016) Endosome-ER contacts control actin nucleation and retromer function through VAP-dependent regulation of PI4P. Cell 166, 408–423 10.1016/j.cell.2016.06.037 27419871 PMC4963242

[68] Wang, Y., Mousley, C.J., Lete, M.G. and Bankaitis, V.A. (2019) An equal opportunity collaboration between lipid metabolism and proteins in the control of membrane trafficking in the trans-Golgi and endosomal systems. Curr. Opin. Cell Biol. 59, 58–72 10.1016/j.ceb.2019.03.012 31039522 PMC7198063

[69] Yan, D., Mayranpaa, M.I., Wong, J., Perttila, J., Lehto, M., Jauhiainen, M. et al. (2008) OSBP-related protein 8 (ORP8) suppresses ABCA1 expression and cholesterol efflux from macrophages. J. Biol. Chem. 283, 332–340 10.1074/jbc.M705313200 17991739

[70] Du, X., Kumar, J., Ferguson, C., Schulz, T.A., Ong, Y.S., Hong, W. et al. (2011) A role for oxysterol-binding protein-related protein 5 in endosomal cholesterol trafficking. J. Cell Biol. 192, 121–135 10.1083/jcb.201004142 21220512 PMC3019559

[71] Ghai, R., Du, X., Wang, H., Dong, J., Ferguson, C., Brown, A.J. et al. (2017) ORP5 and ORP8 bind phosphatidylinositol-4, 5-biphosphate (PtdIns(4,5)P 2) and regulate its level at the plasma membrane. Nat. Commun. 8, 757 10.1038/s41467-017-00861-5 28970484 PMC5624964

[72] Sohn, M., Korzeniowski, M., Zewe, J.P., Wills, R.C., Hammond, G.R.V., Humpolickova, J. et al. (2018) PI(4,5)P2 controls plasma membrane PI4P and PS levels via ORP5/8 recruitment to ER-PM contact sites. J. Cell Biol. 217, 1797–1813 10.1083/jcb.201710095 29472386 PMC5940310

[73] Sohn, M., Ivanova, P., Brown, H.A., Toth, D.J., Varnai, P., Kim, Y.J. et al. (2016) Lenz-Majewski mutations in PTDSS1 affect phosphatidylinositol 4-phosphate metabolism at ER-PM and ER-Golgi junctions. Proc. Natl Acad. Sci. U.S.A. 113, 4314–4319 10.1073/pnas.1525719113 27044099 PMC4843478

[74] Zhong, S., Hsu, F., Stefan, C.J., Wu, X., Patel, A., Cosgrove, M.S. et al. (2012) Allosteric activation of the phosphoinositide phosphatase Sac1 by anionic phospholipids. Biochemistry 51, 3170–3177 10.1021/bi300086c 22452743 PMC3329130

[75] Nishimura, T., Gecht, M., Covino, R., Hummer, G., Surma, M.A., Klose, C. et al. (2019) Osh proteins control nanoscale lipid organization necessary for PI(4,5)P2 synthesis. Mol. Cell 75, 1043–1057.e8 10.1016/j.molcel.2019.06.037 31402097 PMC6739424

[76] Kattan, W.E., Liu, J., Montufar-Solis, D., Liang, H., Brahmendra Barathi, B., van der Hoeven, R. et al. (2021) Components of the phosphatidylserine endoplasmic reticulum to plasma membrane transport mechanism as targets for KRAS inhibition in pancreatic cancer. Proc. Natl Acad. Sci. U.S.A. 118, e2114126118 10.1073/pnas.2114126118 34903667 PMC8713765

[77] Epand, R.M. (2017) Features of the phosphatidylinositol cycle and its role in signal transduction. J. Membr. Biol. 250, 353–366 10.1007/s00232-016-9909-y 27278236

[78] Michell, R.H. (1975) Inositol phospholipids and cell surface receptor function. Biochim. Biophys. Acta 415, 81–47 10.1016/0304-4157(75)90017-9 164246

[79] Amarilio, R., Ramachandran, S., Sabanay, H. and Lev, S. (2005) Differential regulation of endoplasmic reticulum structure through VAP-Nir protein interaction. J. Biol. Chem. 280, 5934–5944 10.1074/jbc.M409566200 15545272

[80] Garner, K., Hunt, A.N., Koster, G., Somerharju, P., Groves, E., Li, M. et al. (2012) Phosphatidylinositol transfer protein, cytoplasmic 1 (PITPNC1) binds and transfers phosphatidic acid. J. Biol. Chem. 287, 32263–32276 10.1074/jbc.M112.375840 22822086 PMC3442557

[81] Kim, S., Kedan, A., Marom, M., Gavert, N., Keinan, O., Selitrennik, M. et al. (2013) The phosphatidylinositol-transfer protein Nir2 binds phosphatidic acid and positively regulates phosphoinositide signalling. EMBO Rep. 14, 891–899 10.1038/embor.2013.113 23897088 PMC3807235

[82] Kim, Y.J., Guzman-Hernandez, M.L., Wisniewski, E. and Balla, T. (2015) Phosphatidylinositol-Phosphatidic acid exchange by Nir2 at ER-PM contact sites maintains phosphoinositide signaling competence. Dev. Cell 33, 549–561 10.1016/j.devcel.2015.04.028 26028218 PMC4476625

[83] Pemberton, J.G., Kim, Y.J., Humpolickova, J., Eisenreichova, A., Sengupta, N., Toth, D.J. et al. (2020) Defining the subcellular distribution and metabolic channeling of phosphatidylinositol. J. Cell Biol. 219 (3) 10.1083/jcb.201906130 PMC7054996 32211894

[84] Zewe, J.P., Miller, A.M., Sangappa, S., Wills, R.C., Goulden, B.D. and Hammond, G.R.V. (2020) Probing the subcellular distribution of phosphatidylinositol reveals a surprising lack at the plasma membrane. J. Cell Biol. 219, e201906127 10.1083/jcb.201906127 32211893 PMC7054989

[85] Chang, C.L. and Liou, J. (2015) Phosphatidylinositol 4,5-bisphosphate homeostasis regulated by Nir2 and Nir3 proteins at endoplasmic reticulum-plasma membrane junctions. J. Biol. Chem. 290, 14289–14301 10.1074/jbc.M114.621375 25887399 PMC4505499

[86] Gatta, A.T., Wong, L.H., Sere, Y.Y., Calderon-Norena, D.M., Cockcroft, S., Menon, A.K. et al. (2015) A new family of StART domain proteins at membrane contact sites has a role in ER-PM sterol transport. eLife 4, e07253 10.7554/eLife.07253 26001273 PMC4463742

[87] Murley, A., Sarsam, R.D., Toulmay, A., Yamada, J., Prinz, W.A. and Nunnari, J. (2015) Ltc1 is an ER-localized sterol transporter and a component of ER-mitochondria and ER-vacuole contacts. J. Cell Biol. 209, 539–548 10.1083/jcb.201502033 25987606 PMC4442815

[88] Naito, T., Ercan, B., Krshnan, L., Triebl, A., Koh, D.H.Z., Wei, F.Y. et al. (2019) Movement of accessible plasma membrane cholesterol by the GRAMD1 lipid transfer protein complex. eLife 8, e51401 10.7554/eLife.51401 31724953 PMC6905856

[89] Ercan, B., Naito, T., Koh, D.H.Z., Dharmawan, D. and Saheki, Y. (2021) Molecular basis of accessible plasma membrane cholesterol recognition by the GRAM domain of GRAMD1b. EMBO J. 40, e106524 10.15252/embj.2020106524 33604931 PMC7957428

[90] Ferrari, A., He, C., Kennelly, J.P., Sandhu, J., Xiao, X., Chi, X. et al. (2020) Aster proteins regulate the accessible cholesterol pool in the plasma membrane. Mol. Cell. Biol. 40, e00255-20 10.1128/MCB.00255-20 32719109 PMC7491948

[91] Sandhu, J., Li, S., Fairall, L., Pfisterer, S.G., Gurnett, J.E., Xiao, X. et al. (2018) Aster proteins facilitate nonvesicular plasma membrane to ER cholesterol transport in mammalian cells. Cell 175, 514–529.e20 10.1016/j.cell.2018.08.033 30220461 PMC6469685

[92] Trinh, M.N., Brown, M.S., Seemann, J., Vale, G., McDonald, J.G., Goldstein, J.L. et al. (2022) Interplay between Asters/GRAMD1s and phosphatidylserine in intermembrane transport of LDL cholesterol. Proc. Natl Acad. Sci. U.S.A. 119, e2120411119 10.1073/pnas.2120411119 34992143 PMC8764668

[93] Wilhelm, L.P., Wendling, C., Vedie, B., Kobayashi, T., Chenard, M.P., Tomasetto, C. et al. (2017) STARD3 mediates endoplasmic reticulum-to-endosome cholesterol transport at membrane contact sites. EMBO J. 36, 1412–1433 10.15252/embj.201695917 28377464 PMC5430228

[94] Acoba, M.G., Senoo, N. and Claypool, S.M. (2020) Phospholipid ebb and flow makes mitochondria go. J. Cell Biol. 219, e202003131 10.1083/jcb.202003131 32614384 PMC7401802

[95] Kim, H., Lee, S., Jun, Y. and Lee, C. (2022) Structural basis for mitoguardin-2 mediated lipid transport at ER-mitochondrial membrane contact sites. Nat. Commun. 13, 3702 10.1038/s41467-022-31462-6 35764626 PMC9239997

[96] Yeo, H.K., Park, T.H., Kim, H.Y., Jang, H., Lee, J., Hwang, G.S. et al. (2021) Phospholipid transfer function of PTPIP51 at mitochondria-associated ER membranes. EMBO Rep. 22, e51323 10.15252/embr.202051323 33938112 PMC8183395

[97] Fairn, G.D., Hermansson, M., Somerharju, P. and Grinstein, S. (2011) Phosphatidylserine is polarized and required for proper Cdc42 localization and for development of cell polarity. Nat. Cell Biol. 13, 1424–1430 10.1038/ncb2351 21964439

[98] Deng, Y., Rivera-Molina, F.E., Toomre, D.K. and Burd, C.G. (2016) Sphingomyelin is sorted at the trans Golgi network into a distinct class of secretory vesicle. Proc. Natl. Acad. Sci. U.S.A. 113, 6677–6682 10.1073/pnas.1602875113 27247384 PMC4914164

